# Editorial: Updates in ocular therapeutics and surgery, volume II

**DOI:** 10.3389/fmed.2024.1370515

**Published:** 2024-02-08

**Authors:** Georgios D. Panos, Horace Massa

**Affiliations:** ^1^Department of Ophthalmology, Queen's Medical Centre, Nottingham University Hospitals, Nottingham, United Kingdom; ^2^Division of Ophthalmology and Visual Sciences, School of Medicine, University of Nottingham, Nottingham, United Kingdom; ^3^Department of Ophthalmology, Geneva University Hospitals, Geneva, Switzerland

**Keywords:** cornea, macular oedema, uveitis, glaucoma, vitreoretinal surgery, machine learning, eyelid surgery, paediatric ophthalmology

## Introduction

It is with great pleasure that we introduce Volume II of “*Updates in Ocular Therapeutics and Surgery*,” building upon the success of its predecessor in the ever-evolving field of ophthalmology. This volume emerges at a pivotal time when the landscape of eye care is being redefined by novel treatments and advanced surgical techniques, particularly for conditions like age-related macular degeneration, glaucoma, and diabetic macular oedema, among others.

Recent technological advancements have revolutionised ocular surgery, making procedures safer, faster, and more precise. This Research Topic aims to highlight these transformative therapeutic and surgical approaches, offering a comprehensive overview of the current state-of-the-art and setting a direction for future research. Each article in this Research Topic not only enriches our understanding but also underscores the rapid progress in ocular medicine.

As we delve into this volume, we invite our readers to explore the significant strides made in treating and understanding ocular diseases, marking another milestone in the journey of ophthalmology.

## Anterior segment

In this Research Topic, we explore a range of groundbreaking studies in the field of anterior segment ocular therapeutics and surgery. Swain and Eliassi-Rad present a five-year retrospective study on selective laser trabeculoplasty (SLT) in glaucoma patients, revealing mixed long-term efficacy with half of the eyes achieving controlled intraocular pressure (IOP), but an increase in glaucoma medications and progression in Humphrey visual field (HVF) parameters.

Cai et al. investigate the efficacy and safety of oral voriconazole (VCZ) as primary treatment for fungal keratitis (FK). Their study demonstrates significant healing and visual acuity improvement in most cases, although larger ulcers and hypopyon were linked to reduced treatment response, highlighting the need for individualised therapeutic approaches.

The global impact of the COVID-19 pandemic on corneal donor tissue harvesting and transplantation is reviewed by Mousavi et al.. They report a worldwide decline in donor tissue volume and elective corneal transplant procedures during lockdowns, with regional variations in the extent of impact, underscoring the pandemic's significant influence on ophthalmic services.

Deshmukh et al. provide an updated review on the management of keratoconus, covering the latest advancements in treatments ranging from corneal cross-linking to gene therapy. The review also discusses the emerging role of artificial intelligence in early detection and management of the condition.

Xu C. et al.'s study on the effects of resveratrol in rat models of corneal allograft rejection (CGR) reveals its potential in prolonging graft survival and reducing inflammation, implicating the PI3K/Akt pathway as a key mechanism.

Onoe et al. compare the outcomes of microhook ab interno trabeculotomy (μLOT) combined with phacoemulsification and iStent inject W implantation in primary open-angle glaucoma patients. Their findings suggest comparable efficacy between the two surgical methods, with significant improvements in IOP and reduced medication requirements.

Finally, Huang et al. explore the relationships between lens diameter (LD) and ocular biometric parameters through ultrasound biomicroscopy. They find that larger LD is associated with elder age, male gender, and larger white-to-white distance, particularly in eyes without extreme myopia, providing valuable insights for personalised surgical planning and visual outcome enhancement.

## Posterior segment

In the domain of posterior segment ocular therapeutics and surgery, this Research Topic presents a series of insightful studies contributing significantly to our understanding and management of various conditions.

Fan S. et al. conduct a systematic review and meta-analysis on the efficacy and safety of a single-dose intravitreal dexamethasone (DEX) implant in treating non-infectious uveitic macular oedema. Their analysis, encompassing 201 eyes, demonstrates notable improvements in visual acuity and central macular thickness with manageable increased intraocular pressure, highlighting the potential of this treatment in uveitic macular oedema.

Shi et al. delve into the outcomes of revision surgery for persistent idiopathic macular hole (PIMH) post-failed primary vitrectomy. The study evaluates the effectiveness of extended internal limiting membrane (ILM) peeling combined with silicone oil (SiO) or air tamponade. Results show promising closure rates, especially for macular holes ≤650 μm in diameter, suggesting the viability of this approach in PIMH treatment.

In a groundbreaking effort, Wang et al. aim to develop a theoretical formula for intraocular lens power calculation in silicone oil-dependent eyes. Testing their formula on 32 patients, they find a strong correlation with actual clinical outcomes, offering a valuable guide for clinicians in selecting appropriate intraocular lenses in these complex cases.

Klaas et al.'s study compares the risk of transient vision loss (TVL) in intravitreal aflibercept injections using a novel prefilled syringe (PFS) against the traditional vial system (VS). Their findings indicate a higher risk associated with the PFS, underscoring the importance of informed consent and careful consideration when using this formulation.

Lastly, Fan W. et al. present a retrospective study aimed at developing a predictive model for elevated intraocular pressure following vitreoretinal surgery with silicone oil tamponade. Employing machine learning techniques, they analyse various predictive factors in over a thousand eyes, culminating in a model with significant accuracy. This model could potentially aid clinicians in anticipating and mitigating the risk of high intraocular pressure postoperatively.

## Adnexa and paediatric ophthalmology

In the Adnexa and Pediatric Ophthalmology field of this Research Topic, we explore diverse and significant advancements in the field. Chaurasia et al. investigate the relationship between eccentric downward eye movement and depth of anaesthesia in paediatric ophthalmic surgeries. Their study, which combines retrospective and prospective data, reveals a notable correlation between the depth of sevoflurane anaesthesia and the occurrence of eccentric downward eye positioning, highlighting the critical need for precise anaesthesia monitoring in paediatric ocular procedures.

Zhou et al. conduct a retrospective analysis to assess the impact of pre-operative axial length on the myopic shift in children undergoing congenital or developmental cataract surgery. The study finds that children with longer pre-operative axial lengths experience slower myopic shifts post-surgery, providing valuable insights for targeted refraction planning in paediatric cataract procedures.

In a pioneering effort, Xu P. et al. develop biomimetic poly(lactic-co-glycolic acid) scaffolds for eyelid reconstruction, aiming to replicate the microstructure of the natural eyelid. Their study introduces three types of scaffolds, including one with azithromycin loading, and demonstrates their efficacy *in vitro* and *in vivo*, suggesting their potential as promising alternatives for eyelid tarsal plate substitutes.

Li et al.'s retrospective study compares the long-term efficacy of botulinum toxin type A (BTXA) injections and surgery in treating acute acquired comitant esotropia. They discover that while both treatments are effective, surgery provides more precise and enduring results. Importantly, the study also finds that patients who respond well to BTXA initially have better long-term outcomes.

Lastly, Song et al. evaluate the effectiveness of optimal pulse technology (OPT) in the treatment of chalazions. Their study shows significant improvements in chalazion size and meibomian gland area following two sessions of OPT treatment. This non-invasive approach also leads to decreased conjunctival congestion, positioning OPT as an effective non-surgical option for adult chalazion treatment.

This Research Topic has traversed a wide spectrum of groundbreaking research and innovative practices in the field of ophthalmology. From the nuanced complexities of anterior and posterior segment treatments to the delicate intricacies of adnexa and paediatric ophthalmology, each article contributes profoundly to our expanding knowledge base. The insights offered here not only reflect the current state-of-the-art but also pave the way for future explorations and advancements. As we close this issue, we extend our heartfelt gratitude to the authors, reviewers, and readers who have contributed to and engaged with these pivotal discussions. It is our collective endeavor that continues to drive the frontiers of ophthalmic research and clinical practice, with the ultimate goal of enhancing patient care and vision health globally.

## Author contributions

GP: Conceptualisation, Data curation, Formal analysis, Funding acquisition, Investigation, Methodology, Project administration, Resources, Software, Supervision, Validation, Visualisation, Writing – original draft, Writing – review & editing. HM: Methodology, Validation, Writing – review & editing.

